# Advances in total glycomic analysis including sialylated sub-glycan isomers by SALSA method

**DOI:** 10.1016/j.bbadva.2025.100144

**Published:** 2025-01-28

**Authors:** Masaki Kurogochi, Chiharu Suzuki, Hisatoshi Hanamatsu, Jun-ichi Furukawa

**Affiliations:** a Institute for Glyco-core Research (iGCORE)**,** Nagoya University**,** Nagoya**,** 464-8601**,** Japan; b Department of Orthopaedic Surgery, Faculty of Medicine and Graduate School of Medicine, Hokkaido University, Sapporo, 060-8638, Japan

**Keywords:** Glycomics, Glycoconjugate, Sialic acid, Mass spectrometry, Automation

## Abstract

All eukaryotic cell surfaces are coated with various types of glycans, which are essential molecules in biological events. In this review, we summarize recent integrated glycomics studies using various biological samples. We introduce an improved sialic acid linkage-specific alkylamidation (SALSA) method for sialylated glycan analysis and an automated glycosphingolipid-glycan preparation system for large-scale glycomic analysis of human plasma/serum. Finally, we explain the importance of integrated glycomics of glycoconjugates through total glycomic analysis of human serum and mouse brain tissue, and discuss prospects for exploring glycans as effective biomarkers of biological phenomena.

## Highlights

• Recent integrated glycomics are reviewed.
• Sialic acid linkage-specific derivatizations of various glycans allow the discrimination of sialylated glycan isomers.
• An automated GSL-glycan preparation system can be performed for large-scale glycomic study by MALDI-TOF MS.
• Insights from integrated glycomics and total glycomics including five major classes of glycans (N-glycans, O-glycans, GSL-glycans, glycosaminoglycans, and free oligosaccharides).

## Introduction

**Fig. 1:**
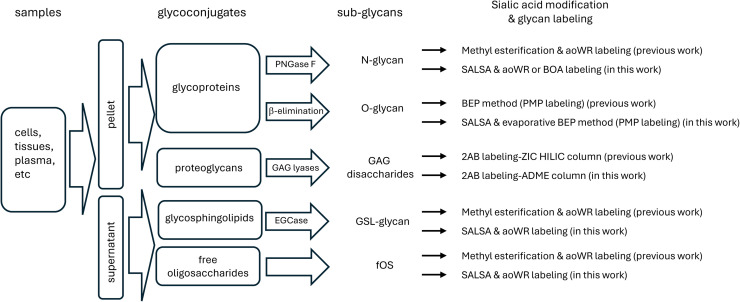
The differences of total glycomics (N-glycans, O-glycans, GAGs, GSL-glycans, and fOSs) between previous work and recent approaches.

Glycans are one of the major components of biomolecules, in addition to nucleic acids, proteins, and lipids. The cell surfaces are covered with a high density of various glycans [1], which link to proteins and lipids to form a variety of glycoconjugates such as glycoproteins, glycolipids, proteoglycans, and glycosylphosphatidylinositol anchor proteins (GPI-APs). Glycans play key roles in many biological processes, especially cell–cell interactions. Therefore, the analysis of glycans is one of the most important areas in the study of glycobiology. The glycome is the entire set of glycans produced or modified in an individual organism, and glycomics is the large-scale study of entire glycomes, similar to genomics and proteomics. A wide variety of glycans (called sub-glycans) are present in biological samples. To analyze these sub-glycomes, it is necessary to cleave the glycans from the glycoconjugates. The methods used to purify and analyze sub-glycomes vary depending on the class of glycoconjugates. For example, when studying the function of a glycosyltransferase gene, it is common to monitor a single glycan class of glycoconjugates through the use of a transgenic or knockout mouse in which a particular gene is targeted, but few whole-glycome analyses have shown whether different classes of glycans derived from various glycoconjugates are affected. Previously, we performed a comprehensive glycomic analysis of five major classes of sub-glycomes derived from glycoproteins, glycosphingolipids (GSLs), free oligosaccharides and proteoglycans, called total glycomic analysis [2]. Streamlined protocols of total glycome analysis are shown in Fig. 1. Total glycomic analysis of 18 human cell lines, including 4 embryonic stem cell lines and 5 induced pluripotent stem cell lines, revealed that cellular sub-glycomes are highly specific to cell type, indicating their utility as unique cellular descriptors. Furthermore, we applied total cellular glycomic analysis not only to identify the known pluripotency biomarkers such as SSEA-3, -4, and -5 (GSL-glycans), as well as considered Tra-1–60 and Tra-1–81 epitope (O-glycans), but also to discover 17 glycans as novel biomarker candidates whose expression levels significantly differs between stem and non-stem cells.

Here, we summarize recent integrated glycomic approaches using various biological samples. Next, we introduce an improved “sialic acid linkage-specific alkylamidation” (SALSA) method for the analysis of all sialylated glycans derived from glycoconjugates and an automated GSL-glycans preparation system for large-scale glycomic analysis of human plasma/serum. Finally, we cover total glycome of human serum and mouse brain tissue by an improved total glycomic analysis adopted the SALSA method (Fig. 1).

## Analytical approaches to integrated glycomics

We first describe some examples of major integrated glycomics conducted before 2018. Gizaw et al. reported N-, O-, and GSL-glycomics of Huntington's disease (HD) transgenic mice [3]. N-glycans were cleaved with Peptide N-Glycosidase F (PNGase F), O-glycans were released by non-reductive β-elimination using ammonium carbamate, and GSL-glycans were released by ozonolysis and alkaline degradation. Each class of glycans was captured on hydrazide beads via chemoselective ligation (glycoblotting method), and carboxylic acids of sialic acid residues were converted to methylesters using 1-methyl-3-*p*-tolyltriazene on the solid phase. Glycans were then released from the beads, labeled with O-benzylhydroxylamine or aminooxy-functionalized tryptophanylarginine methyl ester (aoWR), and analyzed by matrix-assisted laser desorption/ionization (MALDI) - time of flight (TOF) mass spectrometry (MS). In mouse brain tissue, 86 N-glycans, 14 O-glycans, and 6 GSL-glycans were observed. Although the numbers of N- and O-glycans were comparable in mouse serum, more than 20 GSL-glycans were observed. The expression levels of several glycans differed significantly between HD transgenic and control mice. In an integrated glycomic (N- and GSL-glycans) analysis of human Alzheimer's disease (AD) brains, serum, and cerebrospinal fluid (CSF), various glycans in both serum and cerebrospinal fluid (CSF) were analyzed to discover biomarkers [4]. By N-glycomics, 67 N-glycans were detected, and the expression levels of bisect-type N-glycans and multiply branched glycoforms were significantly greater in the AD patient group in both serum and CSF. In addition, the levels of some gangliosides appeared to be altered. In 2016, we reported total glycomic analysis of human and mouse serums. We prepared five sub-glycans based on the glycoblotting method and the alkaline β-elimination with pyrazolone labeling (BEP) method in a similar manner as total cellular glycomics and quantitatively measured them by MALDI-TOF and high-performance liquid chromatography (HPLC) analyses [5]. We quantified 131 serum glycans, including N-glycans, free oligosaccharides (fOSs), glycosaminoglycans (GAGs), O-glycans, and GSL-glycans. In both human and mouse serum, N-glycans were most abundant in the total serum glycome, while fOSs were least abundant. As expected, the diversity of sialic acid (i.e., Neu5Ac vs. Neu5Gc) was the major difference between human and mouse in terms of N- and O-glycosylation, while GSL-glycomic profiles were completely different, even when sialic acid diversity was considered. GSL-glycans in human serum were composed mainly of LacCer (Hex2), GM3 (Hex2Neu5Ac1), Gb3 (Hex3), and Gb4/Lc4 (Hex3HexNAc1), whereas those in mouse serum were composed mainly of GM2 (Hex2HexNAc1Neu5Gc1).

Next, we introduce some examples of integrated glycomics conducted since 2018 (Table 1) [[6], [7], [8], [9], [10], [11], [12], [13], [14], [15], [16], [17], [18], [19], [20], [21], [22], [23], [24], [25], [26], [27], [28], [29], [30]]. In these studies, N-glycans were generally cleaved with PNGase F. The next step is reducing the aldehyde group or labeling it at the reducing end. GSL-glycans are also cleaved by enzymes such as endoglycoceramidase (EGCase) I or II, and then the aldehyde group at the reducing end is similarly reduced or labeled. Free glycans such as fOSs and human milk oligosaccharides (HMOs) can be directly reduced or labeled without enzymatic digestion [14,24,27]. O-Glycans are generally cleaved by chemical digestion, because no enzyme can cleave them comprehensively. The main problems of chemical digestion are the low cleavage efficiency of O-glycans and the peeling reaction, which causes glycan decomposition [31]. O-Glycans are cleaved from proteins by means of β-elimination reactions under non-aqueous or aqueous alkaline conditions, and subsequent processing varies. Therefore, analytical data for O-glycans differ depending on the method. Consequently, when data analyzed by different methods are compared, attention must be paid to the glycan profile, which reflects the amount of glycans and the ratio of decomposed products [32,33]. N-Glycans, O-glycans, GSL-glycans, free glycans are measured by MALDI-TOF MS or LC-electrospray ionization (ESI) MS with high sensitivity and high resolution. GAGs are complex linear polysaccharides consisting of repeating disaccharide units, which are extremely difficult to analyze owing to their negative charge, polydispersity, and structural heterogeneity. Therefore, GAGs are digested with enzymes (heparinase I and III, hyaluronidase SD, and chondroitinase ABC) to prepare GAG disaccharides. Sulfated disaccharides are easily detected as multivalent ions due to their acidic charge, so it is difficult to quantify ionization; therefore, GAG disaccharides are labeled with fluorescent tags and quantified by HPLC [2,5,9,20,30].

**Table 1:** Integrated glycomic studies since 2018.

In 2018, Benktander et al. reported the analysis of three classes of glycans from glycoconjugates derived from human fluids and mouse tissue [6]. In O-glycan analysis, O-glycopeptides were first prepared from glycoproteins with the use of pronase and then permethylated under non-aqueous alkaline conditions to simultaneously cleave O-glycans from glycopeptides. They measured all classes of glycans by MALDI MS and LC-MS using a porous graphitic carbon (PGF) column. Chengjian et al. analyzed N- and O-glycans of ovomucin in human seminal plasma [8]. Both glycans were released by ammonia/NaOH-catalysis and derivatized with 1-phenyl-3-methyl-5-pyrazolone (PMP) by the one-pot release and labeling termed OPRAL. The N- and O-glycans were simultaneously measured by LC-ESI MS using an amide-HILIC column.

In 2019, we reported total cellular glycomics during chondrocyte differentiation [9]. Total cellular glycome alterations are closely associated with chondrocyte hypertrophy. In addition, expressions of genes related to glycan biosynthesis and metabolic processes significantly correlate with glycan alterations.

In 2020, Li et al. reported integrated structural N-glycomic, O-glycomic, and glycosphingolipidomic analysis in cells and tissues [13]. They analyzed glycolipids, including GSLs, without EGCase digestion. Glycolipids were subjected to Folch extraction and then C8 solid-phase-extraction. Enriched glycolipids were measured by C18-nano LC-ESI MS. Site-specific glycoproteomic analysis was also performed using protein digestion. Yu et al. analyzed the N- and O-glycans of proteins in human saliva from patients with Type 2 diabetes mellitus and healthy volunteers [16]. The sialic acids on the isolated glycoproteins were linkage-specific amidated, and then the N-glycans were cleaved with PNGase F. The O-glycan acids were released by oxidation with NaClO. The released N-/O-linked glycans were purified through Hypercarb SPE cartridges and then measured by MALDI-TOF MS. Lac​NAc-containing N- and O-glycans were elevated in patients with Type 2 diabetes.

In 2021, we measured N-, O-, and GSL-glycans in human serum and CSF according to ABO blood types [19]. The integrated glycomic analysis revealed that blood group-specific glyco-antigens are predominantly present on GSLs in serum/plasma. Novel ABO blood group-specific fOSs containing lacto-N-difucotetraose were identified in serum and CSF.

In 2024, we reported that articular cartilage core fucosylation regulates tissue resilience in osteoarthritis (OA) [30]. We also performed total tissue glycomic analysis of human OA cartilage and revealed that most complex/hybrid-type N-glycans were modified with core fucose. Ortega-Rodriguez et al. reported an integrated glycomic method using etanercept and Chinese hamster ovary (CHO) cells. N-Glycans and O-glycopeptides were prepared by PNGase F and proteinase K, respectively. After purification, the enriched N-glycan/O- glycopeptide mixture underwent reduction and permethylation [29]. Both N- and O-glycans were measured simultaneously by MALDI-TOF MS.

Recently, many reports have been published on integrated glycomics, and there is a trend to evaluate samples by analyzing multiple sub-glycans in glycoconjugates. However, there are no examples of GAG analysis in integrated glycomics other than by our group, and analysis of GPI anchors has not been published, which indicates it is difficult to comprehensively analyze glycoconjugates.

## Sialic acid derivatization of N-glycans, GSL-glycans, fOSs, and O-glycans to distinguish sialyl glycan isomers

Sialic acids, such as Neu5Ac, are acidic monosaccharides linked at the non-reducing ends of N-, O-, and GSL-glycans via α2,3-, α2,6-, and α2,8-linkages. Different linkages are associated with various biological events such as cell-cell interactions and cell signaling. Although sialic acids commonly have α-glycosidic linkages, they are more labile than other glycosidic bonds, resulting in loss of terminal sialic acid residues due to the presence of free carboxy groups during MS analysis, which is called the in-source decay phenomenon. To stabilize sialic acid residues, many modifications or salt formation methods of their carboxy groups have been reported. We developed the SALSA method to stabilize and distinguish sialylated glycan isomers by MS analysis [34]. In GSL- and N-glycomic analysis, each glycan is released from glycoproteins and glycolipids by enzymatic digestion [35,36] and captured on beads with a high density of hydrazide groups. The carboxy groups are derivatized by the SALSA method on the solid phase (glycoblotting and SALSA), followed by release and labeling of glycans by the imine exchange reaction for MS analysis. SALSA derivatization allows discrimination of different linkages of sialic acid and high sensitivity in MS owing to sialic acid protection. For example, 14 sialylated GSL-glycans, including Hex2HexNAc1Neu5Ac1, Hex3HexNAc1Neu5Ac1, Hex3HexNAc1Neu5Ac2, Hex4HexNAc2Neu5Ac1, and Hex3HexNAc3Neu5Ac3, are classified into two groups of α2,3- and α2,6-linked sialic acid isomers on the basis of the SphinGOMap database (https://www.lipidmaps.org/resources/pathways/sphingomap_imp). We detected Hex3HexNAc1Neu5Ac1 and Hex4HexNAc2Neu5Ac1 as both α2,3- and α2,6-linked sialic acids in human serum by aminolysis-SALSA [34]. In addition, as the SALSA method does not retain intramolecular lactone intermediates by lactone ring-opening aminolysis, it provides more quantitative measurement than the methyl esterification method.

**Fig. 2:**
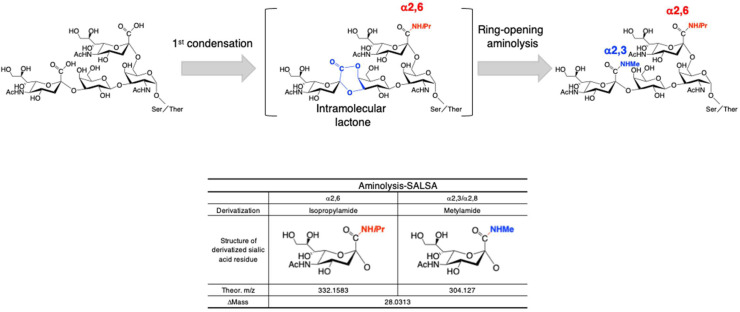
Chemical pathways of sialic acid linkage-specific alkylamidation by lactone ring-opening aminolysis (aminolysis-SALSA).

Recently, we attempted to analyze sialyl O-glycans by the SALSA method. O-Glycans were cleaved from proteins by non-reductive β-elimination to add hydroxylamine [37] and purified as for N- and GSL-glycans. However, SALSA could not be performed because of undesired intramolecular lactone formation between the carboxy group of α2,6-linked sialic acid and the hydroxyl group at the C-5 position of acyclic GalNAc at the reducing end, which resulted in conversion of both isopropylamide and methylamide [26]. To avoid undesired intramolecular lactone formation, we performed SALSA modification as same as N- and GSL-glycans before cleavage of the O-glycans from proteins as shown in Fig. 2. However, the methyl amide groups of α2,3-linked sialic acids were completely degraded by the general reductive β-elimination method. In sharp contrast, sialyl O-glycans on glycoproteins directly derivatized with SALSA were efficiently cleaved from proteins by the evaporative BEP method without degrading the amidation under mild alkaline conditions, allowing quantitative analysis of sialyl O-glycan isomers [38]. We could analyze all sialylated N-, GSL-, and O-glycans to discriminate sialyl glycan isomers by the SALSA method. The development and improvement of various glycomic analyses should lead to a new era of total glycomics.

## Automated glycan preparation for MALDI-TOF MS analysis

The Human Glycome Atlas Project was launched in April 2023, spearheaded by three Japanese institutes: the Tokai National Higher Education and Research System (Nagoya University, Gifu University), the National Institutes of Natural Sciences, and Soka University. In this project, we aim to construct a knowledge base of human glycans and glycoproteins. The creation of a large-scale human glycome catalog (total human serum/plasma glycome) and data storage in TOHSA is one of the most important objectives [39]. To evaluate and verify the associations between glycomics/glycoproteomics and clinical factors, a large number of samples are required, and their analyses must be highly reproducible and reliable. Currently, large-scale glycomic analysis of N-glycans is being performed, and several high-throughput measurement techniques have been applied to measure glycans, including capillary electrophoresis with laser-induced fluorescence detection, ultra-performance LC with fluorescence detection, nano-LC-MS using a PGF column, and MALDI-TOF MS. The development of automated sample preparation platforms is essential for large-scale glycomic analysis. Examples can automate sample preparation with 2-aminobenzamide labeling [40], permethylating N-glycans in a 96-well plate format [41], and preparing N-glycans by linkage-specific sialic acid esterification [42].

**Fig. 3:**
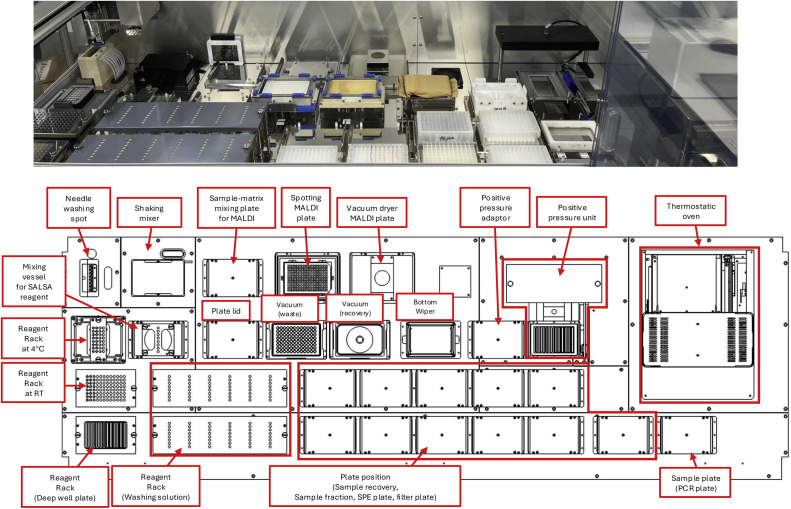
Arrangement of automated GSL-glycan purification system.

As described above, we established protocols for analytical methods including the SALSA method to evaluate N-, GSL- and O-glycans in human serum/plasma and constructed automated glycan preparation systems for glycomic analysis. The automated GSL-glycan purification system is shown in Fig. 3. This system is compatible with various 96-well plates and uses an 8-channel multi-syringe to dispense samples of 1 to 2500 µL. The thermostatic oven can control the temperature from room temperature to 100 °C for enzymatic sample preparation and chemical glycan ligation. Vigorous mixing on a plate shaker promotes the SALSA reaction. In addition, purified and derivatized GSL-glycans in serum/plasma are mixed with matrix, spotted on a MALDI plate, and dried in vacuum.

Although this system is almost the same as the automated N-glycan purification system [43], it is newly equipped with a positive pressure unit. The total amount of GSL-glycans in human serum/plasma is ∼1 % of that of N-glycans; therefore, excess glycoproteins should be removed from plasma/serum to obtain a clean mass spectrum of GSL-glycans. The positive pressure unit allows automated protein removal from samples by filter plates instead of manual ethanol precipitation with centrifugation. aoWR-labeled GSL-glycans can be further purified by solid-phase extraction within it. This system can complete all processes for GSL-glycan preparation from 96 serum/plasma samples within 16 h.

## Total glycomics

In the analysis of GAG disaccharides, by changing the HPLC conditions from using a ZIC-HILIC column with the ability to perform ion-exchange separation to using a reversed-phase column with an adamantyl group, we shortened the analysis time and improved analytical durability [44] (Fig. 1). The protocol of analytical method for GAGs could also be improved towards an automation system, and total glycomic methods were basically established.

**Fig. 4:**
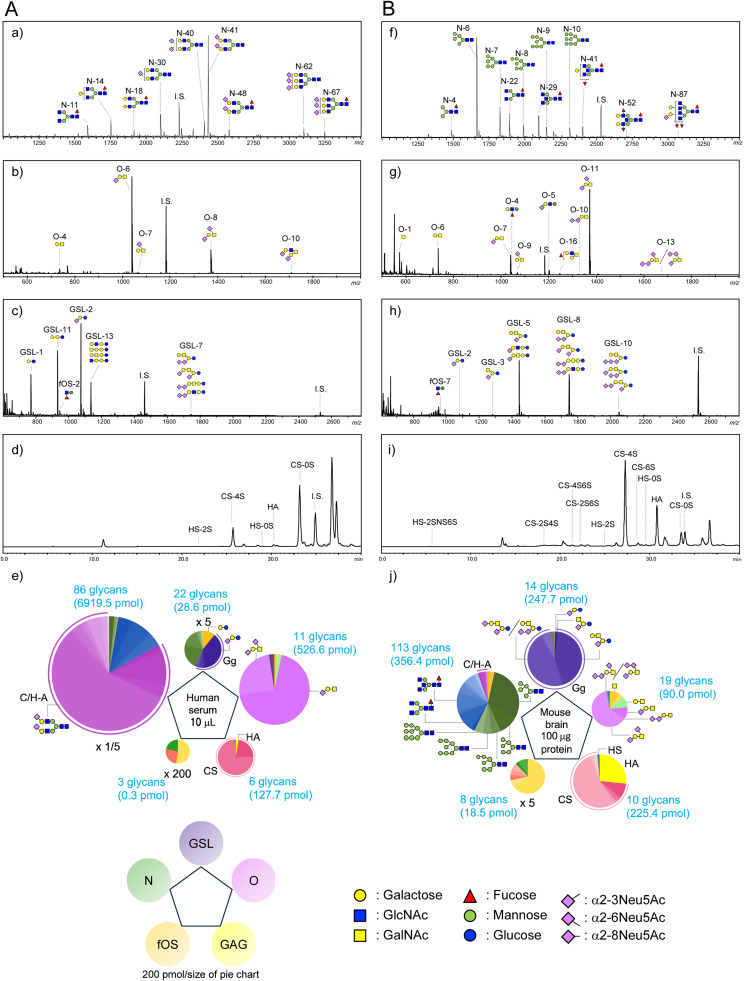
Representative MALDI-TOF MS spectra, HPLC chromatograms, and pentagonal notations showing the glycomic profiles obtained from (A) human serum and (B) mouse brain tissue. MALDI-TOF MS spectra of (a) BOA labeled N-glycans, (b) PMP labeled O-glycans, (c) aoWR labeled glycosphingolipid-glycans and free oligosaccharides. HPLC chromatograms of (e) 2-AB labeled glycosaminoglycans derived from human serum. (f) aoWR labeled N-glycans, (g) PMP labeled O-glycans, (h) aoWR labeled glycosphingolipid-glycans and free oligosaccharides. HPLC chromatograms of (i) 2-AB labeled glycosaminoglycans. Comprehensive glycome profiles (N-glycans, GSL-glycans, O-glycans, GAGs, and fOSs) shown as (e, j) pentagonal notations. The sizes of the pie chart represent the amount of each sub-glycan, and each color represents the different glycan structures. The pie size for N-glycan content in human serum decreased by 5-fold, whereas the pie sizes for GSL and fOS content in human serum increased by 5- and 200-fold, respectively. The pie size for fOS in mouse brain increased by 5-fold.

We measured five classes of glycans derived from human serum and mouse brain tissue. MALDI-TOF-MS spectra and HPLC chromatograms derived from human serum are shown in Fig. 4A a-d. Total serum glycomic profiles are shown as pentagonal notations (Fig. 4e). Pie charts at the vertices of pentagonal notation indicate the expression profiles of GSL-glycans, O-glycans, GAGs, fOSs, and N-glycans. The size of each represents the quantitative amount of each sub-glycan in 10 μL of serum, and the colors indicate the glycan structures. The expression level of each sub-glycan is similar to that in our previous report [5]. With the ability to distinguish sialic acid linkage patterns, we have greatly expanded the number of known N-glycans in human serum, from 46 in 2016 to 86, and that of O-glycans from 5 to 11; identified 22 GSL-glycans; and added structural information [5]. Including 6 GAGs and 3 fOS glycans, 128 glycans are known in human serum (Table 2a), an increase of 40 % on our previous report.

**Table 2:** Glycans in total glycomics of (a) human serum and (b) mouse brain tissue.

We also performed total glycomic analysis of mouse brain tissue as shown in Fig. 4B f-i. After ethanol precipitation of brain homogenate, we analyzed the proteinaceous pellet and the supernatant (including GSLs and fOSs) by the analytical protocol for serum/plasma with minor modifications. Expression of individual GSL-glycans was highest among classes (Fig. 4B; Table 2b). In total, 164 glycans were detected in mouse brain, comprising 113 N-glycans, 14 GSL-glycans, 19 O-glycans, 10 GAG disaccharides, and 8 fOSs. The proportion of GSL-glycans was significantly higher in mouse brain tissue, indicating that the brain is rich in GSLs that are important for the formation of brain tissue. O-glycan analysis detected di- and tri-sialyl T with α2,8-linked sialic acid, which are not present in serum. A high proportion of highly sulfated disaccharides derived from GAGs (CS-2S4S, CS-2S6S, CS-4S6S, and HS-2SNS6S) were characteristic of glycoconjugates in mouse brain.

## Conclusions

This review covers studies that analyzed two or more classes of glycans derived from glycoconjugates in biological samples. An increase in the number of reports of integrated glycomics indicates the development of glycomics with technological advances and increasing interest in the roles of glycoconjugates in biological phenomena. The most common combination of integrated glycomic analysis is N- and O-glycans, which are generated by post-translational modifications of glycoproteins. For example, N-acetylglucosaminyltransferase V (GnT-V), a paralog of GnT-Vb (IX), is expressed in many tissues, including brain. GnT-V and GnT-Vb(IX) were thought to synthesize 1,6-branched N-glycans and O-mannosyl-branched glycans, respectively. However, analysis of N- and O-glycans in GnT-V and GnT-Vb(IX) transgenic mice suggests that GnT-V is involved not only in N-glycan branching, but also in the synthesis of O-mannosyl-branched glycans. It was thought that GnT-V synthesizes only 1,6-branched N-glycans, while GnT-Vb (IX) synthesizes only O-mannosyl branched glycans. However, in mice with homo- and hetero-knockout of GnT-V and GnT-Vb (IX) genes, GnT-V could synthesize O-mannosyl branched glycans in O-glycans. This indicates the importance of N- and O-glycan analysis when studying the function of glycosyltransferases during neuromorphogenesis [45]. The development of integrated glycomic analysis will accelerate the elucidation of various sub-glycan networks regulated by numerous glycosyltransferases.

Other integrated glycomics analyses studied free glycans, such as HMOs. Examples include the use of EGCase to analyze GSL-glycans. Such analyses are also needed to investigate the functions of glycan-related enzymes that may act on a variety of glycoconjugates.

Currently, most reports of glycans in glycoconjugates associated with disease concern N-glycans, followed by GAGs and dystroglycan [1,46]. There is no report of integrated glycomics including GAG analysis, likely because it is difficult to quantitatively analyze GAGs using a mass spectrometer.

PNGase F, which is commercially available and widely used for cleavage of N-glycans, has contributed to progress in N-glycomics. EGCase I and II, also commercially available, are used for GSL-glycomic analysis in many studies. However, GSLs possessing the β-galactosyl ceramide linkage, sulfatide, and GM4 ganglioside, are resistant to enzymatic hydrolysis [47], so these enzymes have limitations on substrate specificity. In GAG analysis, we cannot use keratanase to digest keratan sulfate due to the difficulty of simultaneous GAG separation. Although these issues need to be resolved, we expect that such glycomics will become more widespread.

Recent technological advances in sialic acid linkage-specific derivatization of glycans have provided useful information and improved the sensitivity of sialylated glycan detection by mass spectrometry, especially in MALDI-TOF MS [48]. The direct SALSA method combined with BEP method can be applied to O-glycomic analysis for the first time and is very effective for quantitative analysis of sialylated O-glycan isomers [38].

In large-scale cohort studies, it is essential that processes of sample pretreatment be automated. Several sample preparation devices have been developed for high-throughput N-glycomic analysis over several thousand samples [49]. Our group is developing instruments for the automated preparation of samples of five sub-glycans and has developed instruments for the automated preparation of N- and GSL-glycans. These instruments can sequentially perform enzyme reactions, sialic acid linkage-specific derivatization, glycan purification, solid-phase extraction, and spotting onto MALDI plates, processing up to 96 samples.

Integrated glycomics showed that Lec1 CHO cells lacking N-acetylglucosaminyl transferase I activity caused not only reasonable complex-type N-glycan alterations, but also unexpected increases of other O-linked glycans, GAGs, and GSL glycans [2]. We emphasize the importance of comprehensive glycomics to elucidate different sub-glycan networks, and hope that integrated serum/plasma glycomic analysis including GAGs and glycosphingolipids will become a method to identify new disease biomarkers.

## CRediT authorship contribution statement

**Masaki Kurogochi:** Writing – review & editing, Writing – original draft, Visualization, Funding acquisition, Data curation. **Chiharu Suzuki:** Writing – review & editing, Visualization, Validation. **Hisatoshi Hanamatsu:** Writing – review & editing, Writing – original draft, Visualization, Validation, Funding acquisition, Data curation. **Jun-ichi Furukawa:** Writing – review & editing, Writing – original draft, Project administration, Funding acquisition, Data curation, Conceptualization.

## Declaration of competing interest

The authors declare that they have no known competing financial interests or personal relationships that could have appeared to influence the work reported in this paper.

## Data Availability

Data will be made available on request.
